# Reducing Physical Violence Toward Primary School Students With Disabilities

**DOI:** 10.1016/j.jadohealth.2017.09.004

**Published:** 2018-03

**Authors:** Karen Devries, Hannah Kuper, Louise Knight, Elizabeth Allen, Nambusi Kyegombe, Lena Morgon Banks, Susan Kelly, Dipak Naker

**Affiliations:** a London School of Hygiene and Tropical Medicine, London, United Kingdom; b Raising Voices, Kampala, Uganda

**Keywords:** Violence, Physical abuse, Children, Adolescents, Disabilities, Uganda, School-based interventions

## Abstract

**Purpose:**

We tested whether the Good School Toolkit reduces physical violence from peers and school staff toward students with and without disabilities in Ugandan primary schools.

**Methods:**

We conducted a cluster randomized controlled trial, with data collected via cross-sectional surveys in 2012 and 2014. Forty-two primary schools in Luwero District, Uganda, were randomly assigned to receive the Good School Toolkit for 18 months, or to a waitlisted control group. The primary outcome was past week physical violence from school staff, measured by primary 5, 6, and 7 students' (aged 11–14 years) self-reports using the International Society for the Prevention of Child Abuse and Neglect Child Abuse Screening Tool-Child Institutional. Disability was assessed through the six Short Set Washington Group questions on functioning. Analyses were by intention to treat.

**Results:**

At endline, 53% of control group students with no functional difficulties reported violence from peers or school staff, versus 84% of students with a disability. Prevalence of past week physical violence from school staff was lower in intervention schools than in the control schools after the intervention, in students with no functional difficulties (adjusted odds ratio [aOR] = .41, 95% confidence interval [CI .26–.65]), students with some functional difficulties (aOR = .36, 95% CI .21–.63), and students with disabilities (aOR = .29, 95% CI .14–.59). The intervention also reduced violence from peers in young adolescents, with no evidence of a difference in effect by disability status.

**Conclusions:**

The Good School Toolkit is an effective intervention to reduce violence perpetrated by peers and school staff against young adolescents with disabilities in Ugandan primary schools.

**Implications and Contribution**

No school-based interventions to reduce violence against children with disabilities have been rigorously evaluated in low- and middle-income countries. The Good School Toolkit, a universally targeted school-based intervention to reduce physical violence from school staff to primary school students, is effective in reducing violence against young adolescents with disabilities.

Alt-text: Unlabelled box

Every year, between 500 million and 1.5 billion children and adolescents around the world experience violence [1]. Violence has a long-lasting negative impact on children, including on their physical and mental health [2]. Recent evidence from national surveys in several East African countries shows that violence, in particular physical violence, from school staff is an extremely common form of violence against children under 18, with roughly half of children reporting exposure in Kenya and Tanzania [3], [4]. Patterns are likely to be similar in Uganda, although data are lacking. One survey of over 1,400 children found that >80% had experienced caning and slapping by teachers [5]. In Tanzania, and other similar contexts, reasons teachers give for using physical punishment include: to maintain class discipline and that corporal punishment is perceived to contribute to a student's good academic achievement [6], [7].

Peer violence is also common at school, with between 25% and 63% of students reporting bullying in the past 30 days across 8 African countries [8]. However, rigorously evaluated interventions to reduce physical violence from school staff in low-income and middle-income settings have been almost entirely absent until recently, and antibullying interventions in similar settings are also few [9].

Marginalized children and adolescents may be particularly vulnerable to experiencing violence, and those with disabilities are potentially an important group. Globally, 93 million children under 18 are estimated to be living with a disability, most of whom live in low- and middle-income countries (LMICs) [10]. A recent systematic review showed that children with disabilities are three to four times more likely to be victims of violence than their peers without disabilities [11]. A similar trend is found among adults with disabilities [12]. However, both reviews highlighted issues with the quality of the studies. Furthermore, few data are available from LMICs, and these are mostly qualitative in nature [13], [14], [15], [16].

There is an urgent need for the identification of effective interventions that reduce violence perpetrated against people with disabilities, particularly for LMICs [17]. The Good School Toolkit, by Ugandan nongovernmental organization, Raising Voices, is a complex behavioral intervention that aims to foster change of operational culture at the school level (publicly available at www.raisingvoices.org). The intervention targets multiple levels within the schools with multilayered training, processes, and school-led activities involving head teachers, administration, teachers, and the students themselves.

The Toolkit draws the transtheoretical model [18] and contains behavioral change techniques that have been shown to be effective in a variety of fields [19]. The Toolkit materials consist of books, booklets, posters, and facilitation guides for about 60 different activities. These activities are implemented over six steps in schools and are related to creating a better learning environment, respecting each other, creating opportunities for students to participate in decision-making processes, understanding power relationships, using nonviolent discipline, improving classroom management techniques, and promoting responsive school governance (Figure 1). “Step One: Your Team & Network” is the precontemplation phase, where schools identify key protagonists at school and create their Good School Committee to build school-wide support for the process. “Step Two: Preparing for Change” is the contemplation phase, where baseline measurements on each schools' starting point and school leaders cultivate interest among parents, the community, and local education officials. “Step Three: Good Teachers & Teaching” is the preparing for change phase, where a school-wide reflection on teacher-student relationships provides a renewed sense of teacher roles, increased professional support, and new approaches for positive student engagement. “Step Four: Positive Discipline” is the action phase, where schools reflect on how violence manifests and establish a new school culture by exploring positive disciplinary methods to create students who believe in themselves. “Step Five: Good Learning Environment” is the maintenance phase, where schools reflect on what a good learning environment looks like and work with all stakeholders to foster a psychological sense of safety and inclusion. “Step Six: Good Administration & the Future” is where gains are consolidated, and the work of the preceding steps is celebrated through reflection and transfer of leadership to the school administration.

**Figure 1:**
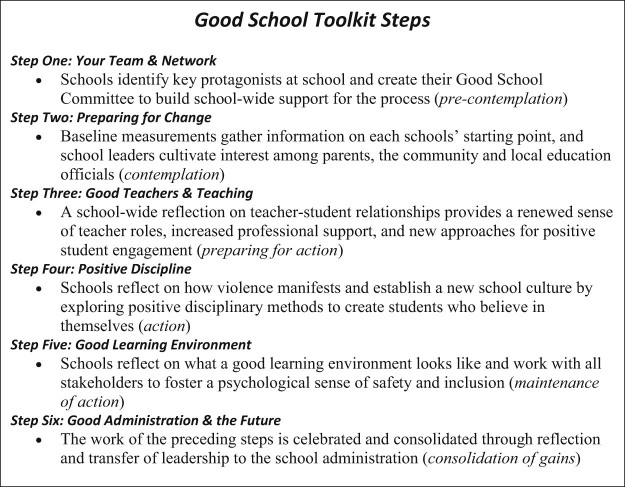
A summary of the Good School Toolkit implementation steps.

These school-led activities are coordinated by two lead teacher “protagonists” and two student representatives in each school. The protagonists and head teachers receive training at program initiation. The schools receive one-on-one support visits (average of two per school term) and phone calls from Raising Voices staff throughout implementation. The schools are encouraged to self-monitor their progress and are also monitored by Raising Voices staff on a monthly basis. Schools received implementation support from Raising Voices over an 18 month period.

A recent cluster randomized controlled trial has shown that this intervention reduced past week physical violence from school staff toward students in Ugandan primary schools by 42% (corresponding to an odds ratio [OR] = .40, 95% confidence interval [CI] .26–.64, *p* < .0001) [20]. It has not yet been assessed whether the intervention was also effective for adolescents with disabilities. This is important as preliminary data from the baseline survey showed that young adolescents with disabilities were more likely to report experiencing violence from school staff [21].

In this paper, we use data from the Good Schools Study, conducted in a representative sample of larger primary schools in Luwero District, Uganda. We examine (1)the prevalence of violence against young adolescents by disability status, (2) the association between disability and experience of physical, sexual, and emotional violence from school staff and peers; (3) which types of impairments are most associated with physical, sexual, and emotional violence; (4) whether young adolescents with disabilities were more or less likely than nondisabled young adolescents to disclose their experiences of violence (and therefore be able to access child protection mechanisms), and who they disclose to; (5) whether young adolescents with disabilities were aware of and able to participate in the Good Schools Toolkit intervention to the same degree as their nondisabled counterparts; and (6) whether the Good School Toolkit was as effective at reducing violence against young adolescents with a disability as young adolescents without a disability.

## Methods

The Good Schools Study (registered at clinicaltrials.gov, NCT01678846) included a cluster-randomized controlled trial in 42 primary schools in Luwero District, Uganda. The study was approved by the London School of Hygiene & Tropical Medicine Ethics Committee (6183) and the Uganda National Council for Science and Technology (SS2520).

Recruitment and randomization are described in detail elsewhere [20]. Briefly, 42 schools were randomly selected from a list of all larger primary schools in Luwero District, and randomly allocated to receive either the Good School Toolkit with implementation support from Raising Voices, or to a wait-list control group. The intervention was implemented over 18 months, between September or October, 2012 and April or May, 2014. Due to the nature of the intervention, participants and those collecting data should be considered unmasked to allocation.

All head teachers agreed for their schools to participate in the study, and cross-sectional baseline and endline surveys were conducted at schools in June or July, 2012 and June or July, 2014, respectively. Parents were notified and could opt their children out, but young adolescents themselves provided consent. Up-to-date lists of all Primary (P) 5, 6, and 7 students (aged about 11–14 years) were obtained from each school, and a simple random sample of up to 130 P5, 6, and 7 students who agreed to participate were then administered surveys individually. If there were fewer than 130 P5–7 students in a school, all were invited for interview. Local interviewers attended 3-week training on interviewing techniques delivered by researchers from the London School of Hygiene and Tropical Medicine and Raising Voices staff. Training included working through scenarios, role-playing, piloting, developing skills required for interviewing children; being nonjudgmental and how to build rapport; reasonable interview adjustments for students with sight, hearing, or other functional difficulties; full consent processes; and referral procedures. The interviewer-administered questionnaire data were collected using a survey programmed into tablet computers with algorithms designed to eliminate erroneous skips. Interviews were conducted face-to-face in a private space where students could be seen but not overheard.

All students who could speak Luganda or English and who were deemed by interviewers to be able to understand the consent procedures were eligible. All young participants were offered counseling, and young adolescents were informed during the consent process that their details might be passed on to child protective services if there were concerns related to violence or other mental health difficulties disclosed by the child [22], [23]. The criteria for referral are fully described elsewhere, but included disclosure of severe and/or recent physical or sexual violence, or less severe or recent physical or sexual violence plus mental health difficulties (measured by scores on the Strengths and Difficulties Questionnaire [24]) [22], [23].

All measures were pretested for understanding and piloted before use, and are outlined in Annex 1. All variables were measured at the level of individual student participants. Disability was measured in the baseline cross-sectional survey using a six-item list to assess functional impairment. In the endline cross-sectional survey, we used the short set Washington Group questions, which consists of six questions assessing functional limitations in six different domains (seeing, hearing, walking, remembering/concentrating, self-care, communicating) at four different levels of difficulty (no, some, a lot, cannot do). These questions are endorsed by the UN for the measurement of disability [25], and are widely used, including in Uganda [26] and many other African countries [27]. These were translated into Luganda and cognitively tested to ensure understanding. Students were classified as having no functional difficulty and having some difficulty in one domain or disability (i.e., a lot of difficulty or more in one domain or some difficulty in at least two domains).

The primary outcome was past week physical violence from a school staff member, self-reported by students according to the International Society for the Prevention of Child Abuse and Neglect Screening Tool—Child Institutional (ICAST-CI). The reliability and construct validity for the ICAST-CI were initially established in four countries, and the instrument has since been translated into 20 languages and used extensively in multicountry research [28], [29], [30]. Instruments were translated where necessary, and some items and time frames for recall were added to the ICAST to capture the Ugandan context. In addition to physical violence from school staff, other forms of violence measured included emotional and sexual violence from school staff and peers, physical violence from peers, and injuries as a result of physical or sexual violence from school staff. Violence outcome variables are modeled as binary (presence of any act of violence vs. absence of all acts over a given time frame), similar to other international studies on violence exposure [31].

Exposure to the intervention was measured using a score constructed based on responses to 10 questions that were asked to students during the endline survey. Individual students' positive responses to questions were counted to produce the student exposure score (0–10). Each question was a statement related to various aspects of the Toolkit intervention, for example: “I have participated in a meeting/Any activity organized by the Good Schools pupils committee” and “My school has written classroom rules and regulations for how pupils should behave.” Whether young adolescents met criteria to be referred to child protection, and whether they had previously disclosed experiences of violence were measured using binary variables. Referral criteria are described elsewhere [22], [23].

All analyses were done in Stata/IC 13.1 (StataCorp, Houston, TX). We used a combination of data from our baseline and endline cross-sectional survey and from control and intervention schools, depending on the research question addressed in each analysis (specified under each table). Analysis was done with individual-level student data. For each analysis, we accounted for clustering of the data at the school level using mixed-effects regression models.

To examine whether the effects of the intervention differed by disability status, we did an intention-to-treat analysis using data from our cross-sectional follow-up survey (Table 4). We fit mixed-effects regression models, adjusted for covariates specified a priori: baseline school-level means of past week physical violence from school staff (the outcome, modeled as a continuous variable at the school level), students' sex, and their school's location (urban or rural). Models with and without an interaction term for study arm and disability status were fit and compared using a likelihood ratio test. *p* values for the likelihood ratio test under .05 were interpreted as evidence of a significant interaction effect, that is, a difference in the effect of the intervention by disability status of the students (Table 4). ORs and 95% CIs represent the intervention effect within each disability subgroup; however, the CIs around each OR in this subgroup analysis should not be overinterpreted.

## Results

Overall, 5.8% of students reported a disability in our endline 2014 survey (Table 1) and 16.9% reported “some” difficulty in one functional domain. The most common difficulty reported was with memory/concentration, followed by sight. The intervention and control arms were well balanced with respect to the characteristics of the young adolescents, including prevalence of disability (Annex 2). The young adolescents with disabilities were more likely to board at school than the young adolescents without disabilities, and there were slightly more boarding students in the control arm.

**Table 1:** Prevalence of different forms of disability

	Total	Control	Intervention
Prevalence	N (%)	N (%)	N (%)
No functional difficulties with any domain	2,956 (77.4)	1,517 (79.9)	1,439 (74.9)
Some functional difficulty with one domain	644 (16.9)	278 (14.6)	366 (19.1)
Disability	220 (5.8)	104 (5.5)	116 (6.0)
Some functional difficulty or disability in the following domains:			
Sight	155 (4.1)	68 (3.6)	87 (4.5)
Hearing	102 (2.7)	43 (2.3)	59 (3.1)
Movement	102 (2.7)	39 (2.1)	63 (3.3)
Memory/concentration	559 (14.6)	259 (13.7)	300 (15.6)
Self-care	44 (1.2)	20 (1.1)	24 (1.2)
Communication	103 (2.7)	44 (2.3)	59 (3.1)

At follow-up, using full data set (intervention and control schools).

Table 2 shows the prevalence of violence reported by disability status in the absence of any intervention (i.e., in the control group at follow-up). The majority of students reported experiencing some form of violence at school, and school staff were key perpetrators. For almost every form of violence—physical, sexual, and emotional violence by staff or peers—students who have some functional difficulties or a disability were more likely to be victimized versus students who report no functional difficulties in any domain.

**Table 2:** Forms of violence reported by children, by disability status

	No functional difficulties in any domain	Some functional difficulty in one domain	Disability	
Prevalence, past week	N = 1,517 N, %	N = 278 N, %	N = 104 N, %	*p*
Total school violence	811 (53.5)	179 (64.4)	87 (83.7)	<.001
Staff				
Any violence	718 (47.3)	161 (57.9)	74 (71.2)	<.001
Physical violence	694 (45.8)	158 (56.8)	72 (69.2)	<.001
Emotional violence	125 (8.2)	35 (12.6)	19 (18.3)	.002
Sexual violence	9 (.6)	0 (0)	4 (3.9)	.004
Any injury	367 (26.8)	80 (30.5)	43 (44.3)	.004
Moderate injury	81 (5.9)	14 (5.3)	10 (10.3)	.213
Severe injury	5 (.4)	0 (0)	2 (2.1)	.042
Peers				
Any violence	306 (20.2)	73 (26.3)	56 (53.9)	<.001
Physical violence	119 (7.8)	30 (10.8)	33 (31.8)	<.001
Emotional violence	232 (15.3)	51 (18.4)	39 (37.5)	<.001
Sexual violence	7 (.5)	2 (.7)	0 (0)	.827

Control schools only at follow-up.

Table 3 shows the experience of different forms of violence for students who had at least some functional difficulties or a disability in each impairment category compared with those who report no functional difficulties, in the absence of any intervention. “Total school violence,” “any violence from peers,” and “physical violence from peers” were more common across all domains of difficulty compared with young adolescents with no functional difficulties. Young adolescents with difficulties in the domains of self-care or communication were more likely to report sexual violence and injuries from school staff compared with young adolescents with no functional difficulties, although this relationship was less apparent for other domains of functional difficulty.

**Table 3:** Odds of violence outcomes by functional difficulty type

	Sight	Hearing	Mobility	Memory/ concentration	Self-care	Communication
Outcome, past week	OR (95% CI)	OR (95% CI)	OR (95% CI)	OR (95% CI)	OR (95% CI)	OR (95% CI)
Total school violence	2.83 (1.40–5.71)	3.29 (1.42–7.62)	2.22 (1.10–4.45)	2.06 (1.62–2.62)	2.61 (1.08–6.29)	3.92 (1.81–8.47)
Staff						
Any violence	2.04 (1.08–3.84)	2.31 (1.34–3.98)	1.44 (.76–2.75)	1.80 (1.41–2.30)	2.60 (.92–7.36)	4.33 (2.10–8.91)
Physical violence	2.17 (1.16–4.06)	2.21 (1.33–3.68)	1.39 (.77–2.49)	1.80 (1.42–2.26)	2.77 (.98–7.83)	4.61 (2.27–9.37)
Emotional violence	1.70 (.95–3.05)	2.55 (1.22–5.29)	1.64 (.63–4.24)	1.68 (1.23–2.29)	1.97 (.62–6.24)	4.18 (2.44–7.15)
Sexual violence	5.1 (.95–27.07)	3.99 (.42–38.2)	4.41 (.44–43.79)	1.96 (.45–8.50)	18.61 (2.08–123.69)	7.98 (1.39–45.87)
Any injury	1.68 (.86–3.29)	.77 (.30–1.95)	1.37 (.60–3.10)	1.53 (1.11–2.10)	3.07 (1.53–6.18)	2.73 (1.39–5.38)
Moderate injury	1.08 (.43–2.70)	.82 (.17–3.88)	.45 (.05–3.84)	1.25 (.60–2.57)	3.41 (1.08–10.78)	2.65 (1.14–6.16)
Severe injury	4.40 (1.04–18.6)	—	7.8 (.78–77.97)	1.11 (.28–4.44)	17.06 (3.50–83.11)	13.65 (4.34–42.96)
Peers						
Any violence	2.94 (1.72–5.01)	3.44 (1.79–6.61)	2.47 (1.56–3.92)	2.04 (1.53–2.72)	3.96 (1.71–9.15)	3.30 (2.06–5.27)
Physical violence	3.32 (2.03–5.45)	4.03 (1.90–8.60)	4.05 (2.29–7.17)	2.08 (1.35–3.22)	6.33 (2.29–17.50)	6.08 (3.72–9.93)
Emotional violence	1.70 (.78–3.72)	2.67 (1.44–4.97)	2.46 (1.37–4.43)	1.82 (1.32–2.50)	2.37 (.84–6.70)	2.08 (1.01–4.28)
Sexual violence	3.22 (.40–25.94)	5.13 (.58–45.9)	—	—	—	—

ORs are unadjusted odds ratios from logistic regression models comparing people who had some functional difficulties or disability (grouped) in each impairment category versus those who report no functional difficulties, using data from control schools only at follow-up.

CI = confidence interval.

In the absence of any intervention, young adolescents with disabilities were more likely to meet the criteria for referral to child protective services, versus those who reported some functional difficulties and those who reported no functional difficulties (Annex 3). This indicates that as a group, young adolescents with disabilities experienced more severe abuse and had higher levels of mental health difficulties. Of those young adolescents who met referral criteria, there were no statistically significant differences in whether they had previously disclosed to another person, with just less than one quarter of students reporting that they had previously told someone about their experience. Of those who did disclose, the most common person to disclose to was a parent. Just over half of students who had previously disclosed to someone reported that disclosure had helped them; this did not differ by disability status.

We tested whether young adolescents with some functional difficulties and those with disabilities were able to access and participate in the Good Schools Toolkit to the same degree as young adolescents without any functional difficulties, by comparing an intervention exposure score across groups of students in intervention schools only. Young adolescents with no functional difficulties reported a median exposure score of 6 (interquartile range [IQR] 3–9); those with some functional difficulties reported a median exposure score of 7 (IQR 3–9), and those with a disability reported a median exposure score of 6 (IQR 3–8). There was a statistically significant difference across groups (Somers *d* test, *p* = .016); however, the difference in score does not have a clear direction and was very small in practice.

The Good Schools Toolkit is successful in reducing a range of different forms of violence from staff and peers toward students (Table 4), including among students who report no functional difficulties, those who report some difficulty in one domain, and those who report a disability (for the primary outcome, “any violence from staff in past week,” the aORs were .42 (.27–.67), .40 (.23–.69), and .27 (.13–.56), respectively). We did not find any evidence of statistically significant differences in effects of the intervention between the three student groups, nor any suggestion of nonsignificant trends which would imply that the Toolkit is less effective for students with disabilities. These findings indicate that the Good School Toolkit intervention can also be considered effective for reducing violence from staff and peers toward students with some functional difficulties or disabilities.

**Table 4:** Effect of the intervention

	No functional difficulties in any domain			Some functional difficulties in one domain			Disability			LR test
Outcome	C (N = 1,517)	I (N = 1,439)	aOR	C (N = 278)	I (N = 366)	aOR	C (N = 104)	I (N = 116)	aOR	*p*
Any violence, staff or peers, past week	811 (53.5)	544 (37.8)	.47 (.31–.71)	179 (64.4)	161 (44.0)	.41 (.25–.67)	87 (83.7)	68 (58.6)	.22 (.10–.48)	.077
Any violence, staff or peers, past term	1,274 (84.0)	946 (65.7)	.32 (.18–.58)	250 (89.9)	524 (69.4)	.22 (.11–.47)	92 (88.5)	94 (81.0)	.47 (.18–1.26)	.223
Any staff violence, past week	718 (47.3)	443 (30.8)	.43 (.27–.67)	161 (57.9)	138 (37.7)	.40 (.23–.69)	74 (71.2)	53 (45.7)	.27 (.13–.56)	.342
Any violence, staff, past term	1,215 (80.1)	865 (60.1)	.33 (.19–.56)	240 (86.3)	231 (63.1)	.23 (.12–.46)	88 (84.6)	89 (76.7)	.49 (.20–1.19)	.173
Physical violence from staff, past week	694 (45.8)	416 (28.9)	.41 (.26–.65)	158 (56.8)	127 (34.7)	.36 (.21–.63)	72 (69.2)	52 (44.8)	.29 (.14–.59)	.427
Physical violence from staff, past term	1,207 (79.6)	847 (58.9)	.32 (.18–.54)	233 (83.8)	224 (61.2)	.26 (.13–.50)	88 (84.6)	86 (74.1)	.42 (.18–1.02)	.481
Any peer violence, past week	306 (20.2)	231 (16.1)	.70 (.53–1.03)	73 (26.3)	70 (19.1)	.70 (.44–1.12)	56 (53.9)	36 (31.0)	.37 (.20–.70)	.073
Any peer violence, past term	496 (32.7)	387 (26.9)	.72 (.52–1.00)	114 (41.0)	120 (32.8)	.69 (.44–1.07)	67 (64.4)	51 (44.0)	.39 (.21–.73)	.117

ORs are adjusted for baseline school mean of variable, *p* value is from likelihood ratio (LR) test comparing model with and without interaction across study arm and disability; past term analysis is adjusted for past week of that variable at baseline (past term not measured at baseline); using full dataset at follow-up.

aOR = adjusted odds ratio; C = control; CI = confidence interval; I = intervention.

## Discussion

The Good School Toolkit was effective at reducing levels of violence from staff and peers toward student with some functional difficulties, and students with disabilities, similar to results among students without any functional difficulties or disabilities. We also showed that in the absence of any intervention, young adolescents with disabilities had an increased risk of experiencing violence in school, compared with young adolescents without disabilities, even within this context of high levels of violence reported by young adolescents overall. The high vulnerability of young adolescents with disabilities to violence was evident across all domains of functional difficulty, but most noticeably for young adolescents with difficulties in self-care or communication. Young adolescents with disabilities were not more or less likely than other young adolescents to disclose previous experience of violence to others.

### Comparison with other studies

Comparable studies testing an intervention to reduce violence against children with disabilities in an LMIC setting are lacking [17], [32]. A systematic review identified 10 studies assessing the effectiveness of interventions to prevent and respond to violence against persons with disabilities [17]. One was conducted in South Africa, whereas the remainder was in high-income countries, and only two included children. All were judged to have a high risk of bias.

A systematic review of 17 studies reported a pooled prevalence estimate of 26.7% (95% CI 13.8–42.1) for reported violence among children with disabilities, including 20.4% reporting physical violence and 13.7% sexual violence [11]. Our prevalence estimates are, therefore, far higher, although the studies in the review showed a high level of variability in reported violence and were all conducted in high-income settings. Furthermore, we used a comprehensive assessment of violence which may have elicited more reports of violence. The systematic review indicated that children with mental or intellectual impairments were particularly vulnerable to violence, which was also documented in a systematic review of violence among adults with disabilities [12]. This tallies with our findings of greater risk of violence among young adolescents with communication or self-care difficulties. We also note that the prevalence of memory and difficulty concentrating was substantially higher than other types of functional difficulties. In this sample, nearly half of all students report eating less than 3 meals the previous day, which may partially account for the high number of students reporting difficulties with memory and concentration.

Few studies have investigated the reasons why children with disabilities are more vulnerable to violence. Possible reasons include a higher level of stigma and discrimination against children with disabilities, lack of support for carers, lower physical and emotional defenses, and communication barriers limiting reporting of violence [11], [12]. It is also speculated that children with disabilities are more likely to be in situations of vulnerability, as is exemplified by the high proportion of the young adolescents with disabilities in this study who were boarders at school. Boarders may experience greater risk of violence due to lack of parental supervision, type or severity of the disability experienced, or other reasons. More research is needed in this area.

The National Census in Uganda in 2006 collected data on disability using the Washington Group Short Set. They estimated the prevalence of “some” difficulty or more in at least one domain at 12.5% for 10- to 14-year-olds [33], which is lower than those from the current study of 16.9% in 11- to 14-year-olds. The census collected the data predominantly from the household head, whereas in this study the children were interviewed directly, which may account for some of the discrepancy. Data on type of disability in adolescents were lacking in Uganda, but in Cameroon it was listed as most likely to be due to difficulties with memory and learning, as it was in this study [34].

### Strengths and limitations

This was a large study, which employed internationally validated questionnaires for assessment of both violence and disability, and which used the gold standard method of a randomized controlled trial for assessing impact of the intervention. We asked about both shorter (past week) and longer (past term) recall periods and consider consistency in responses when interpreting our results. Student responses may have been more reliable over the shorter period; however, numbers of violent acts experienced may be too low to facilitate statistical analysis. Over the longer period, recall may be poorer but higher numbers of acts may have accrued. The study was conducted in the Luwero District which has a mixture of urban and rural communities and demographic characteristics that are broadly similar to the rest of Uganda.

There are also important limitations. Participants and those collecting data were unmasked to allocation, given the nature of the intervention; this may have introduced bias. However, it is likely that young adolescents exposed to the intervention would have felt more able to disclose violence, rather than less. In other words, any bias would likely have been in the opposite direction to the intervention effect, making our results conservative. We did not follow students individually, so we used school-level data to adjust for baseline characteristics. This may have resulted in residual confounding.

Disability at baseline was measured using a single question with multiple response options, and included domains of sight, hearing, mobility, speech, and whether or not students had epilepsy. At follow-up, we used the Washington Group Short Set questions. However, this does not affect our analysis as our main trial comparison makes use of cross-sectional data at follow-up (rather than comparing change in individuals over time), and uses baseline data to adjust for any school-level differences (i.e., the baseline disability variables are not part of the main trial analysis). The Washington Group Short Set questions were developed for people aged 5+  and have undergone extensive cognitive and field testing [25]. These questions are widely used, and have been recommended for use by several UN and other bilateral agencies. They have not been specifically validated against objectively measured functional status, nor has there been specific testing among adolescents. Furthermore, they rely on self-report and so may under- or overestimate the prevalence of objectively measured functional limitations, although there is a lack of evidence on the extent to which this may occur. It is not possible to rule out that the association between vulnerability to violence and disability may in some instances be bidirectional, for instance, with difficulties in concentrating resulting from ongoing exposure to violence.

The study sample was limited to young adolescents at school, although it is known that many children with disabilities are not included in education [35]. Our results should not be interpreted as generalizable to out of school young adolescents with disabilities. Similarly, we have not explored the effects of the intervention on other violence young adolescents may be experiencing outside of school.

### Implications

The Good School Toolkit, a universal intervention aimed at preventing violence perpetrated against children attending school, was also effective for students with disabilities. There was no evidence that young adolescents with disabilities found it more difficult to access the intervention. The Toolkit represents a highly promising strategy that could be widely used to reduce violence against young adolescents with disabilities at schools.

Further research is needed around specifically targeted interventions to prevent violence against children with disabilities, given their high levels of reported violence even after the intervention. Similarly, further research is needed to investigate who are the most common perpetrators of different forms of violence toward children with disabilities who are not attending school, and what interventions may be effective for reducing violence for these children. Effective interventions may include parenting programs [36], as children globally are also vulnerable to experiencing physical and other forms of violence at home [37]. There were low levels of disclosure of experiences of violence among both young adolescents with disabilities and those without, and this is an important area to be addressed. Interventions are also needed to increase referral and inclusion in child protection services. This could include screening of children with disabilities for violence signals [38] and training of teachers, health and social workers, on the vulnerability of children with disabilities to violence.

The Good School Toolkit is an effective intervention to reduce violence perpetrated by school staff and peers against young adolescents with disabilities in Ugandan primary schools.

## Funding Sources

The Good Schools Study was funded by the Joint Global Health Trials Scheme (MRC, DfID, and Wellcome Trust, MR/L004321/1) to K. Devries, and the Hewlett Foundation to Dipak Naker. This analysis was funded by Plan International (to K. Devries).

## Supplementary Data

Annex 1 Measures. Annex 1

Annex 2 Distribution of demographic factors at baseline. Annex 2

Annex 3 Previous disclosure and seeking help. Annex 3
