# Supplementary material for: Reducing Physical Violence Toward Primary School Students With Disabilities
Source: J Adolesc Health. 2018 Mar;62(3):303–10. doi: 10.1016/j.jadohealth.2017.09.004 (PMC5817160; doi:10.1016/j.jadohealth.2017.09.004)
Supplement: Annex 1 — Measures. [file mmc1.docx]

Annex 1. Measures

| Variable name | Instrument, Items | Coding |
| --- | --- | --- |
| School staff violence |  |  |
| Physical violence, severe physical violence*  Time frame: past week, past school term, ever | Has a school staff member: hurt you or caused pain to you? Slapped you with a hand on your face or head as punishment? Slapped you with a hand on your arm or hand? Twisted your ear as punishment? Twisted your arm as punishment? Pulled your hair as punishment? Hit you by throwing an object at you? Hit you with a closed fist? Hit you with a stick? Caned you? Kicked you? Knocked you on the head as punishment? Made you dig, slash a field, or do other labour as punishment? Hit your fingers or hands with an object as punishment? Crushed your fingers or hands as punishment? Made you stand /kneel in a way that hurts to punish you? Made you stay outside for example in the heat or rain to punish you? Burnt you as punishment?* Taken your food away from you as punishment? Forced you to do something that was dangerous?* Choked you? Tied you up with a rope or belt at school?* Tried to cut you purposefully with a sharp object?* Severely beat you up?* | Coded 1 if answered yes to any of the items; 0 if answered no to all items. |
| Emotional violence  Time frame: past week, past school term, ever | Has a school staff member: Cursed, insulted, shouted at or humiliated you? Referred to your skin colour/ gender/ religion/ tribe or health problems you have in a hurtful way? Stopped you from being with other children to make you feel bad or lonely*?* Tried to embarrass you because you were an orphan or without a parent? Embarrassed you because you were unable to buy things? Stole or broke or ruined your belongings? Threatened you with bad marks that you didn’t deserve? Accused you of witchcraft? | Coded 1 if answered yes to any of the items; 0 if answered no to all items. |
| Sexual violence  Time frame: past week, past school term, ever | Has a school staff member: Teased you or made sexual comments about your breasts, genitals, buttocks or other body parts? Touched your body in a sexual way or in a way that made you uncomfortable? By “sexual way” we mean touching you on your genitals, breasts or buttocks. Showed you pictures, magazines, or movies of people or children doing sexual things? Made you take your clothes off when it was not for a medical reason? Opened or took their own clothes off in front of you when they should not have done so? Kiss you when you didn’t want to be kissed? Make you touch their genitals, breasts or buttocks when you didn’t want to? Touch your genitals, breasts or buttocks when you didn’t want them to? Give you money/ things to do sexual things? Involve you in making sexual pictures or videos? Threaten or pressure you to have sex or do sexual things with them? Actually make you have sex with them by threatening or pressuring you, or by making you afraid of what they might do? Make you have sex with them by physically forcing you (have sex with you)? | Coded 1 if answered yes to any of the items; 0 if answered no to all items. |
| Any injury (moderate injury*, severe injury **)  Time frame: past week, past school term, ever | You felt pain? You had bruising?* You had swelling?* You were bleeding?* You had cuts?* It was difficult to sit down on your buttocks?* It was difficult to walk?* You lost consciousness, even temporarily?** You suffered a dislocated, sprained, fractured or broken bone?** You had any other serious injury?** You had to get medical attention, for example from the health worker or hospital? ** You had to stay home from school? | Coded 1 if answered yes to any of the items; 0 if answered no to all items. |
| Peer violence |  |  |
| Emotional violence/neglect  Time frame: past week, past school term, ever | Has anyone besides a school staff member: Insulted you, or called you rude or hurtful names? Accused you of witchcraft? Locked you out or made you stay outside? Not given you food?  Perpetrator asked after each act; multiple perpetrators could be mentioned. | Coded 1 if answered yes to any of the items; 0 if answered no to all items. |
| Physical violence  Time frame: past week, past school term, ever | Has anyone besides a school staff member: Twisted your arm or any other body part, slapped you, pushed you or thrown something at you? Punched you, kicked you, or hit you with a closed fist? Hit you with an object, such as a stick or a cane, or whipped you? Cut you with a sharp object or burnt you?  Perpetrator asked after each act; multiple perpetrators could be mentioned. | Coded 1 if answered yes to any of the items; 0 if answered no to all items. |
| Sexual violence  Time frame: past week, past school term, ever | Has anyone besides a school staff member: Disturbed or bothered you by making sexual comments about you? Kissed you, when you did not want them to? Touched your genitals or breasts when you did not want them to, or in a way that made you uncomfortable? Threaten or pressure you to make you do something sexual with them? Make you have sex with them, because they threatened or pressured you? Had sex with you, by physically forcing you?  Perpetrator asked after each act; multiple perpetrators could be mentioned. | Coded 1 if answered yes to any of the items; 0 if answered no to all items. |
| Disability (baseline) | Do you have any mental or physical disability?  Probe: For example, do you have trouble seeing, walking, speaking, fits, or anything else?  Trouble seeing, trouble hearing, trouble walking/with movement, trouble with speech, fits, other? | Any disability was defined as responding ‘yes’ to any item; no disability was defined as responding no to all items. |
| Disability (endline) | The next questions ask about difficulties you may have doing certain activities because of a health problem. Please tell me if you have no difficulty, some difficulty, a lot of difficulty, or you cannot do this at all. Do you have difficulty seeing, even if wearing glasses? Do you have difficulty hearing, even if using a hearing aid? Do you have difficulty walking or climbing steps? Do you have difficulty remembering or concentrating? Do you have difficulty with self-care, such as washing all over or dressing? Using your usual language, do you have difficulty communicating, for example understanding, or being understood? | ‘No difficulties’ was defined as reporting no difficulty to all items.  ‘Some difficulties’ was defined as reporting some difficulty to one item, but not reporting some difficulty with 2 or more items, and not reporting a lot of difficulty or cannot do this at all to any item.  Disability was defined as reporting some difficulty with 2 or more items, or reporting a lot of difficulty or cannot do this at all to any item. |
| Exposure to intervention | My school has a pupils court that is different than the prefects council or discipline committee, My schools has a Good Schools pupils committee , I have participated in a meeting/Any activity organised by the Good Schools pupils committee, My school has written classroom rules and regulations for how pupils should behave, My class participated in making up these written rules, These written rules are displayed in my classroom where pupils can see them, My school has a wall of fame for pupils, My school has a suggestion box where pupils can put ideas, In my school, I have seen a poster or booklet about Good Schools, like this (show materials), In my school, I have participated in a discussion about Good Schools posters or booklets | Coded 1 if answered yes. Total count of yes answers. Score 0-10 with higher score relating to higher exposure to the intervention. |
| Referral to child protection | Whether child was referred to child protective services based on what they disclosed in the survey. Referral was determined according to responses on the survey, outlined elsewhere^19, 20^. | Binary variable, referred or not. |
