# Supplementary material for: Reducing Physical Violence Toward Primary School Students With Disabilities
Source: J Adolesc Health. 2018 Mar;62(3):303–10. doi: 10.1016/j.jadohealth.2017.09.004 (PMC5817160; doi:10.1016/j.jadohealth.2017.09.004)
Supplement: Annex 2 — Distribution of demographic factors at baseline. [file mmc2.docx]

Annex 2. Distribution of demographic factors at baseline

|  | **No disability** |  | **Disability** |  |
| --- | --- | --- | --- | --- |
| **Demographics** | **Control (n=1737)** | **I (n=1693)** | **C (n=142)** | **I (n=129)** |
| Age (years), mean (SD) | 13.0 (1.5) | 13.1 (1.5) | 13.0 (1.3) | 13.1 (1.7) |
| School class |  |  |  |  |
| 5 | 647 (37.2) | 685 (40.4) | 56 (39.4) | 54 (41.9) |
| 6 | 646 (37.1) | 600 (35.4) | 51 (35.9) | 44 (34.1) |
| 7 | 447 (25.7) | 410 (24.2) | 35 (24.7) | 31 (24.0) |
| Male | 46.0 | 48.9 | 50 | 52.7 |
| Eaten 3+ meals yesterday | 830 (47.7) | 800 (47.2) | 58 (40.9) | 58 (45.0) |
| Hours of work each day |  |  |  |  |
| Less than 1h | 730 (42.2) | 583 (34.5) | 58 (41.1) | 52 (40.6) |
| 1-2 h | 725 (41.9) | 276 (15.9) | 55 (39.0) | 52 (40.6) |
| More than 2 h | 276 (15.9) | 290 (17.2) | 28 (20.0) | 24 (18.8) |
| Mode of transport to school |  |  |  |  |
| Other | 56 (3.3) | 68 (4.1) | 6 (4.4) | 9 (7.4) |
| Walking alone | 401 (23.6) | 419 (25.2) | 39 (28.3) | 31 (25.4) |
| Walking with someone you know | 1092 (64.2) | 1083 (65.2) | 69 (50.0) | 70 (57.4) |
| Board at school | 151 (8.9) | 92 (5.5) | 24 (17.4) | 12 (9.8) |
| Absence from school in previous week |  |  |  |  |
| 1 or more days missed | 310 (18.3) | 396 (23.6) | 37 (28.0) | 29 (23.0) |
