# Supplementary material for: Reducing Physical Violence Toward Primary School Students With Disabilities
Source: J Adolesc Health. 2018 Mar;62(3):303–10. doi: 10.1016/j.jadohealth.2017.09.004 (PMC5817160; doi:10.1016/j.jadohealth.2017.09.004)
Supplement: Annex 3 — Previous disclosure and seeking help. [file mmc3.docx]

Annex 3. Previous disclosure and seeking help.

|  | No functional difficulties in any domain | Some functional difficulty in one domain | Disability |  |
| --- | --- | --- | --- | --- |
| **Characteristic** | **N=1517**  **N, %** | **N=278**  **N, %** | **N=104**  **N, %** | **p** |
| Referred to child protection during Good Schools | 371 (24.5) | 79(28.4) | 54(48.1) | <0.001 |
| **Of those referred:** | **N=371** | **N=79** | **N=54** |  |
| Previously disclosed | 88 (23.7) | 16 (20.3) | 15 (27.8) | 0.602 |
| **Disclosed to:** | **N=88** | **N=16** | **N=15** | 0.355 |
| Parent | 66 (75.0%) | 11 (68.8) | 10 (66.7) |  |
| Teacher | 6 (6.8) | 1 (6.3) | 0 (0) |  |
| Friend | 8 (9.1) | 0(0) | 1 (6.7) |  |
| Sibling | 4 (4.6) | 9(6.3) | 1 (6.7) |  |
| Other | 4 (4.6) | 3 (18.8) | 3 (20.0) |  |
| Disclosure helped | 50 (56.8) | 9 (56.3) | 6 (60.0) | 0.971 |

In control group only at follow up
